# Primary explant culture and collagen I substrate enhances corneal endothelial cell morphology

**DOI:** 10.1186/s13104-018-3174-3

**Published:** 2018-01-18

**Authors:** Judith Zavala, María-Dolores Montalvo-Parra, Guillermo-Isaac Guerrero-Ramírez, Carlos-Alberto Rodríguez-Barrientos, Victor Treviño, Jorge E. Valdez-García

**Affiliations:** 1 Tecnológico de Monterrey, Escuela de Medicina y Ciencias de la Salud, Ave. Morones Prieto # 3000, 64710 Monterrey, NL Mexico; 2 Instituto Universitario Fernández-Vega, Fundación de Investigación Oftalmológica, Universidad de Oviedo, Av. Doctores Fernández Vega # 34, 33012 Oviedo, Asturias Spain

**Keywords:** Corneal endothelium, Primary cell culture, Collagen I

## Abstract

**Objectives:**

Corneal endothelial cell (CEC) isolation and harvest aim to produce engineered grafts to solve donor corneal tissue shortage. To yield high amounts of CEC maintaining morphological and molecular characteristics, several isolation and culture conditions are reported. Here, we combined direct explant culture, with three different coating conditions and a two-step media approach to compare confluence efficiency, morphology, and specific molecular markers expression.

**Data description:**

Confluence was reached after 2 weeks in the three coating conditions (Matrigel, collagen I, and in uncoated plates) using a two-step approach (proliferative medium without pituitary extract, followed by stabilizer basal medium). Na/K-ATPase and GPC4 markers were detected by immunocytochemistry while GPC4, CD200, and TJP1 by RT-PCR in the three CEC coating culture conditions. CEC in proliferative medium showed spindle morphology in the three conditions. Polygonal morphology was seen in CEC cultures using basal medium under uncoated and collagen I coated plates. CEC cultured in Matrigel-coated plates remained with spindle morphology in basal medium.

## Objective

Corneal endothelial cell (CEC) isolation and harvest aim to produce engineered grafts to solve donor corneal tissue shortage. To yield high amounts of CEC maintaining morphological and molecular characteristics, several isolation and culture conditions are reported. Isolation by enzymatic digestion using collagenase, dispase, and trypsin–EDTA yields high proliferation rates. However, this treatment can decrease the viability of CEC obtained [1–3]. Culture conditions comprise the use of substrate coated plates and media with different supplementation, including growth factors such as epidermal (EGF), basic fibroblastic (FGFb), neural (NGF), and insulin growth factor (IGF), along with pituitary extract (PE), CaCl_2_, and ascorbic acid among others, which provides different outcomes in terms of morphology and specific molecular markers expression [3].

This work is focused on the generation of tissue grafts for future corneal endothelium engineering [4–9]. Preliminary results showed that CEC can proliferate when isolated by enzymatic digestion and using a previously reported supplemented medium containing PE [5, 6, 9]. However, isolation by enzymatic digestion yielded low amounts of viable cells (data not available). In addition, the use of PE and FBS generates uncertainty about the components needed for the control of cell identity and proliferation. To set the best conditions for isolation and harvesting of CEC of rabbits for tissue engineering purposes, we tested the removal of enzymatic digestion using direct explant culture, the two-step media approach that uses a proliferative media without PE, and three different coating conditions (Matrigel, collagen I and no coated plate). We compared the outcomes in terms of confluence efficiency, morphology, and specific molecular markers expression.

## Data description

### Materials and methods

Six White New Zealand rabbits 3-month-old were sacrificed under general anesthesia with and a lethal intracardiac injection of sodic pentobarbital. A corneoscleral rim excision was made and the conjunctiva was dissected. Lens and aqueous humor were removed and the corneas were obtained. Under sterile conditions, Descemet’s membrane were separated from corneal stroma and rinsed with basal stabilizer medium (SM) containing OptiMEM-I 8% fetal bovine serum (FBS) and 1% antibiotics. Corneal endothelia were peeled off from the Descemet’s membrane and a ~ 5 mm^2^ section was cultured in proliferative medium (PM) containing OptiMEM-I 8% FBS, 20 ng/mL of nerve growth factor (NGF), 5 ng/mL of epidermal growth factor (EGF), 200 µg/L of calcium chloride, 20 µg/mL of ascorbic acid, 0.08% chondroitin sulfate, and antibiotics over Matrigel, collagen I or no coated plate until confluence (~ 90% of the plate showed adherent cells, ~ 15 days). The plates were tripzinised and cultured in SM. Morphological changes were photodocumented. RNA was isolated from CEC cultured in PM and SM. Final point RT-PCRs were made to analyze the expression of the specific CECs markers: glypican-4 (GPC4), tight junction protein 1 (TJP1), and CD200; housekeeping gene glyceraldehyde phosphate dehydrogenase (GAPDH) was used. Electrophoresis of PCR products was performed on a 2% agarose gel and the bands were photodocumented. Immunocytochemistry was performed to analyze the presence of GPC4 (Abcam, ab150517, Cambridge, UK) and Na/K-ATPase (Abcam, ab176163, Cambridge, UK) in CEC cultured in each condition. Images were obtained with a fluorescence inverted microscope. Detailed description of experimental procedures (rabbit euthanasia, corneal dissection, RNA isolation, RT-PCR parameters, and immunocytochemistry) along with manufacturer information of reagents, equipment, and software is provided in the data repository.

### Results

The figure in data file 1 (located in Figshare: 10.6084/m9.figshare.5771484.v1) shows representative results of the morphological changes of CEC cultures. CEC cultured for 7 days in PM over Matrigel, collagen I and uncoated plates showed spindle morphology. After medium was replaced using SM, characteristic polygonal morphology of CEC was clearly observed after 48 h in collagen I and uncoated plates. CEC in Matrigel remained with spindle morphology. After cultivation in SM over Matrigel, collagen I, and uncoated plates, CEC expressed GPC4 and Na/K-ATPase. Positive immunocytochemistry results of GPC4 in CEC cultured in collagen I and uncoated plates and Na/K-ATPase in CEC cultured in Matrigel are shown (data file 2, located in Figshare: 10.6084/m9.figshare.5771496). CEC cultured with PM over Matrigel and uncoated plates, and CEC cultured in SM over collagen I, Matrigel, and uncoated plates expressed GPC4, CD200, TJP1, and GAPDH (data file 3, located in Figshare: 10.6084/m9.figshare.5615449.v9).

In summary, PM (with no pituitary extract) followed by a SM over uncoated and collagen I coated plates promotes CEC proliferation from primary explant, polygonal shape acquisition and the expression of canonical markers of CEC. Matrigel coated plates also promoted CEC proliferation and expression of canonical markers, but failed to allow the acquisition of polygonal shape, which is essential for CEC to be used for corneal engineering purposes.

## Limitations

This experiment aimed to provide evidence of the advantage of isolating corneal endothelial cells from direct explant culture using a two-step approach over collagen I and uncoated plates. Shortcomings that prevented the data to be used as part of a full research paper were:

- Data collection of immunocytochemistry was not possible for all treatments, given the small amount of CEC obtained for each preparation.
- Data collection of CEC markers expression for RT-PCR in cells cultured in collagen I with proliferative medium was not possible given the small amount of RNA obtained.
- It was not possible to obtain fresh rabbit corneas to repeat the experiments.

## Abbreviations

CEC: corneal endothelial cells
GPC4: glypican 4
TJP1: tight junction protein 1
GAPDH: glyceraldehyde-3-phosphate dehydrogenase
